# Clinical Hypothyroidism Following Sistrunk Procedure: A Diagnostic Pitfall

**DOI:** 10.7759/cureus.101234

**Published:** 2026-01-10

**Authors:** Daniela M Soares, Raquel da Inez Correia, Vítor Valente, José Ricardo Brandão, André Couto de Carvalho

**Affiliations:** 1 Department of Endocrinology, Unidade Local de Saúde de Santo António, Porto, PRT; 2 Department of General Surgery, Unidade Local de Saúde de Santo António, Porto, PRT; 3 Department of Pathology, Unidade Local de Saúde de Santo António, Porto, PRT

**Keywords:** ectopic thyroid, hypothyroidism, neck mass, sistrunk procedure, thyroglossal duct cyst

## Abstract

The differential diagnosis of an anterior neck mass is extensive and may encompass benign or malignant tumors. Most cases are due to congenital malformations, inflammation/infection, or neoplastic conditions. A proper diagnostic approach is essential to ensure adequate management and follow-up. We report the case of a 43-year-old woman referred to our outpatient clinic due to a painless anterior neck mass. Initial clinical and imaging assessment was consistent with a thyroglossal duct cyst, and a Sistrunk procedure was proposed. Clinical and imaging findings suggestive of recurrence led to surgical reintervention 23 months later. Unexpectedly, the patient developed primary hypothyroidism postoperatively, prompting revision of the initial diagnosis. Further imaging studies and subsequent multidisciplinary evaluation led to the correct diagnosis of ectopic thyroid. This case emphasizes the importance of comprehensive laboratory and thorough neck imaging evaluation within a multidisciplinary setting in every case prior to any thyroid-related surgical proposals. Such an approach could reduce morbidity and improve clinical outcomes in these patients.

## Introduction

Anterior neck masses are relatively common lesions that include a wide variety of diagnoses, both benign and malignant. Most diagnosis fall into three main categories: (i) malformation or congenital, like brachial cleft cysts, thyroglossal duct cysts or ectopic thyroid, (ii) inflammatory or infectious, such as lymphadenopathies in the context of viral, bacterial or parasitic infections, and (iii) neoplastic, most prevalent in adults and far exceeding any other etiologies in this age group [1].

An ectopic thyroid consists of functioning thyroid tissue found anywhere other than in the thyroid’s usual anatomic site, typically along the pathway of normal embryological thyroid descent from the base of the tongue. It is more common in female patients, and its prevalence ranges from 1:3,000-6,000 in individuals with recognized thyroid dysfunction to 1:200,000-300,000 in the general population, although it may be particularly underdiagnosed due to its frequent asymptomatic presentation [2]. In turn, thyroglossal duct cysts are far more common (prevalence around seven in 100 individuals), similarly presenting as asymptomatic midline cervical masses [3,4].

Timing of onset of symptoms and the presence of cancer risk factors, along with a thorough physical examination focusing on the mass, adjacent structures, and lymph nodes, provide vital clues towards the correct diagnosis of an anterior neck mass [1,5,6]. Patients should undergo further laboratory and imaging studies, such as neck ultrasound (US) (usually the first imaging option) or computed tomography (CT) scans (in case of uncertain etiology or suspicion of malignancy). Other diagnostic considerations using functional thyroid imaging, such as radioisotope scanning (if suspicion of ectopic thyroid tissue after US performance) or fine-needle aspiration (if uncertain etiology or malignancy suspicion), may also be employed [1,3,6]. A proper diagnosis is essential for the correct management and treatment of an ectopic thyroid gland.

We report the case of a female patient referred due to a painless anterior neck mass, whose first evaluation was consistent with a thyroglossal duct cyst; a Sistrunk procedure was initially proposed. Unexpectedly, the patient developed primary hypothyroidism postoperatively, prompting revision of the initial diagnosis.

## Case presentation

A 43-year-old female patient was referred to our Surgery outpatient clinic due to a painless anterior neck mass with several years of evolution and no cervical compressive symptoms. She had no personal or familial history of previous thyroid disease nor previous exposure to cervical radiation, thyroid-interfering medications, or iodinated contrasts. The last available thyroid function was unremarkable (thyroid-stimulating hormone (TSH) 2.15 mUI/L (reference, 0.35-4.94) and free thyroxine (FT4) 13.97 pmol/L (reference, 12.23-20.20)) (Table 1). The last US report at the time of the referral stated the presence of a “thyroid gland of reduced dimensions with loss of morphological configuration and a large cyst measuring 43 mm in the infrahyoid region, compatible with thyroglossal duct cyst”.

**Table 1:** Laboratory findings LT4: levothyroxine; T4: thyroxine; Tg: thyroglobulin; TPO: thyroid peroxidase; TSH: thyroid-stimulating hormone

Test	Baseline	Patient Value (one month after the second surgery)	Patient Value (four months after LT4 initiation)	Reference Range
TSH	2.15	7.89	4.71	0.35-4.94 mUI/L
Free T4	13.97	10.43	13.50	12.23-20.20 pmol/L
Anti-TPO antibody	-	11.3	-	Negative < 34 UI/mL
Anti-Tg antibody	-	17.6	-	Negative < 115 UI/mL

**Figure 1:**
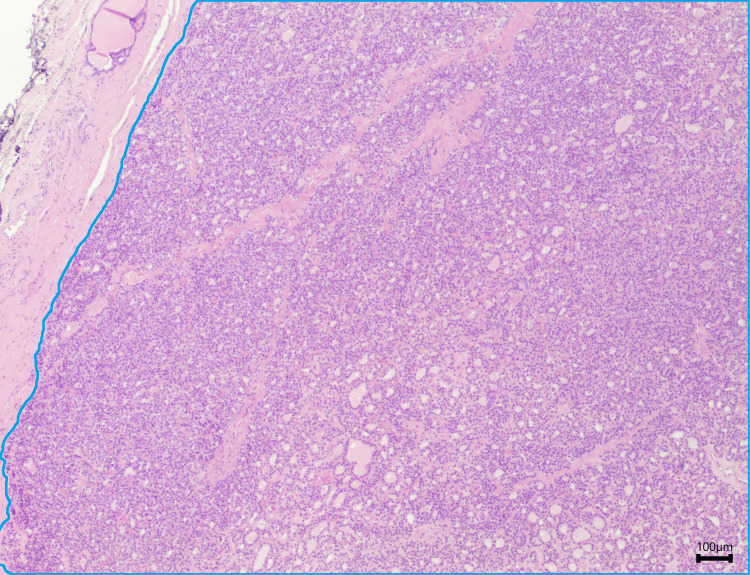
Pathology image from the first surgical neck procedure (Hematoxylin & Eosin, 40x) Thyroid tissue: microfollicles composed of thyroid cells (area within the blue outline).

**Figure 2:**
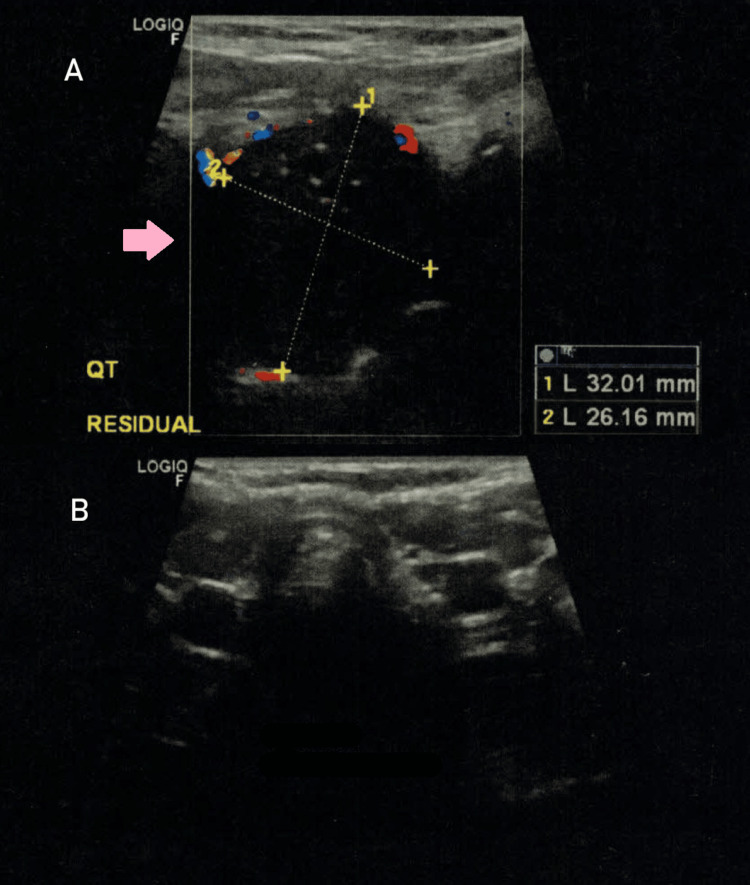
Anterior neck ultrasound (transverse view). (A) Cystic mass of 32x26 mm in the infrahyoid region (pink arrow), with regular edges, dense content and small peripheral calcifications, interpreted as a residual cyst (B) Small (atrophic) thyroid gland with heterogeneous parenchyma

**Figure 3:**
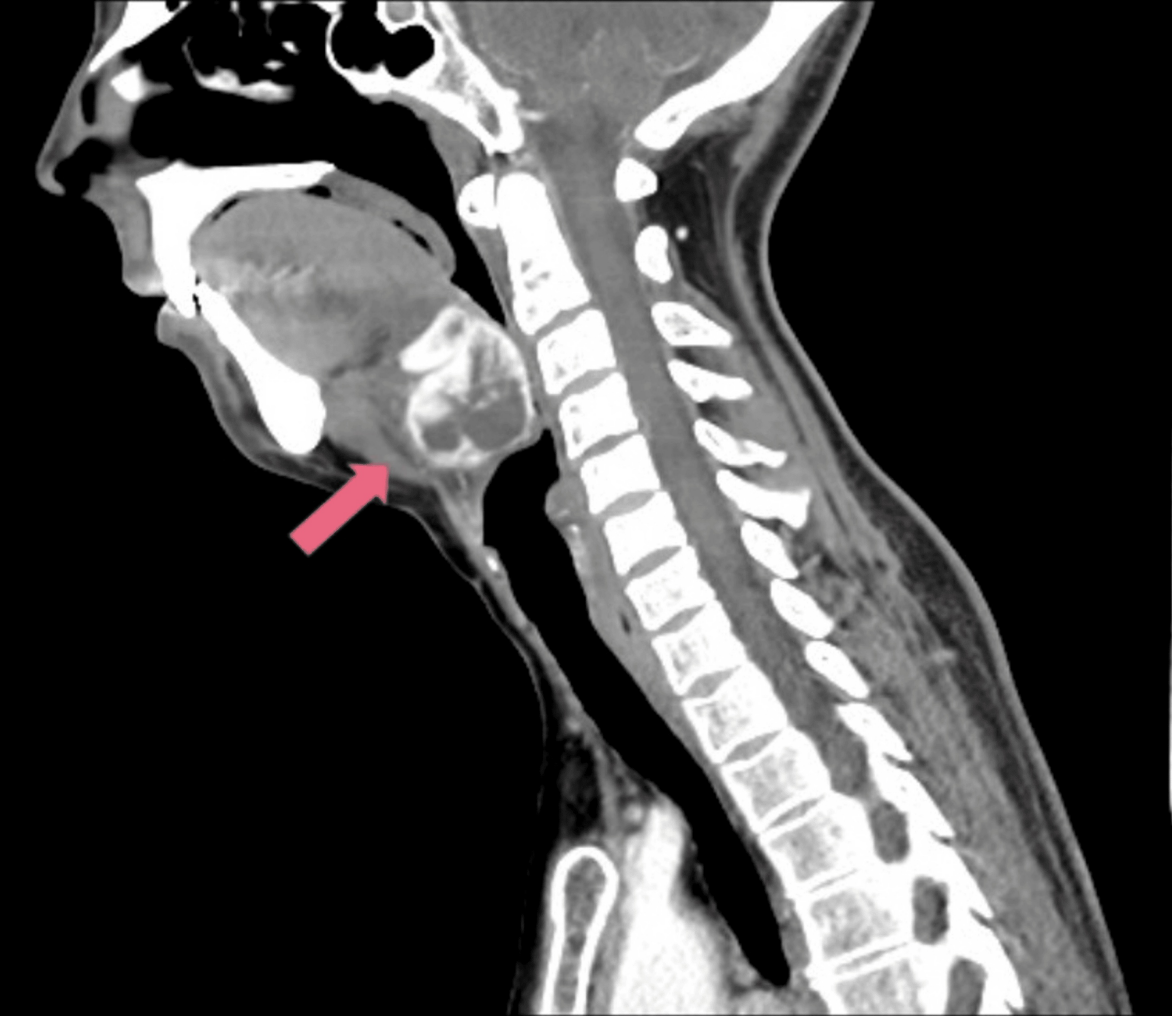
Neck CT scan (sagittal view) Large cystic mass (pink arrow) inferior to the tongue mimicking a thyroglossal duct cyst.

Due to the lesion size, a Sistrunk procedure was proposed, which occurred uneventfully. The pathology report revealed a 5 g fragment of thyroid parenchymal tissue, composed of follicular cells arranged in microfollicles and without nuclear atypia; there was no evidence of cystic epithelial structures or inflammatory processes. These features are also similar to those seen in adenomatous nodules, follicular adenomas, or carcinomas (Figure 1). Considering the clinical perception of local recurrence eight months later, a new neck US was performed, which documented the presence of an eutopic atrophic thyroid gland and a cystic mass of 32x26 mm in the infrahyoid region, with regular edges, dense content, and small peripheral calcifications, interpreted as a residual cyst (Figure 2). A CT scan of the neck was also performed and confirmed the presence of a cystic mass of around 4 cm in the thyroglossal duct, close to the epiglottis, without any references to any eutopic thyroid gland (Figure 3).

**Figure 4:**
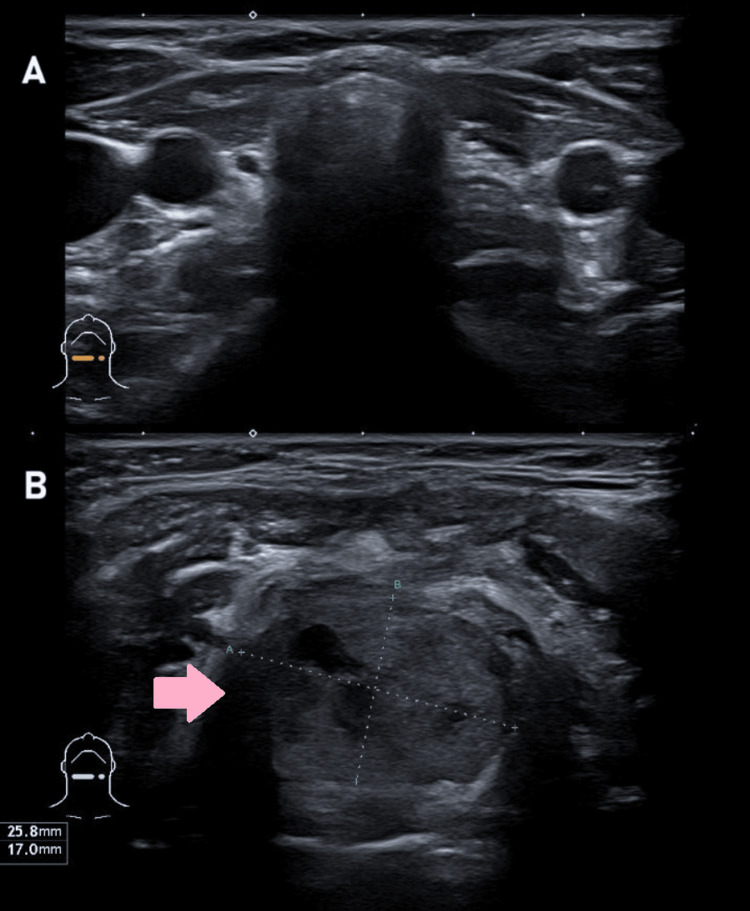
Last anterior neck ultrasound (transverse view). (A) Absence of the thyroid gland in its usual topography (B) A predominantly solid nodular formation in the submental region (pink arrow), 26x17 mm

**Figure 5:**
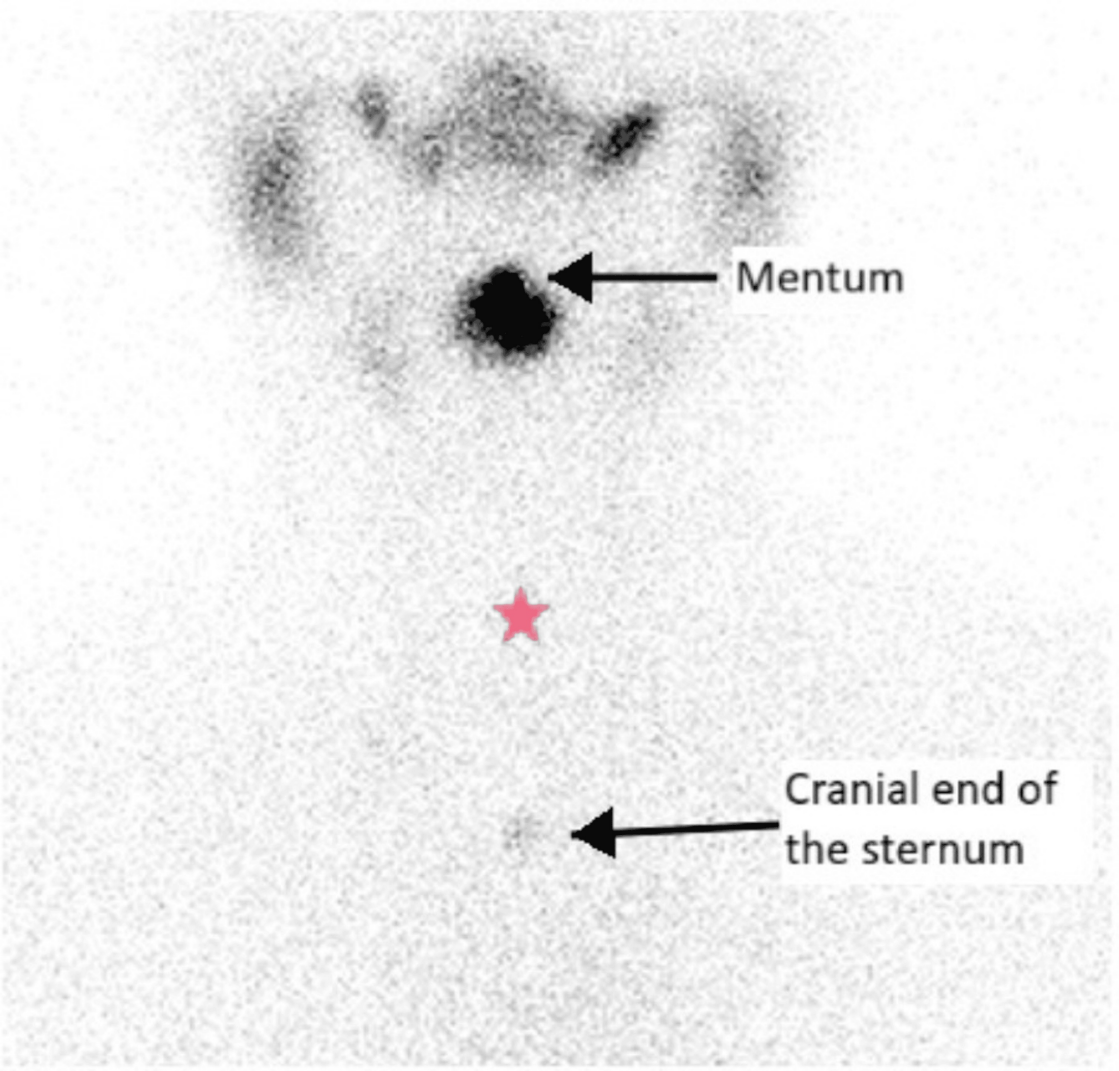
Tc-99m pertechnetate thyroid scintigraphy (coronal view). Increased uptake in the submental region (upper arrow) and absence of uptake in usual thyroid site (pink star).

Based on these findings, reintervention for full mass resection was proposed 23 months after the first surgery. This time, the pathological report framing the thyroglossal duct cyst hypothesis described an 8 g nodular fragment compatible with a follicular tumor of uncertain malignant potential of the thyroglossal duct. Postoperative thyroid function (one month after reintervention) was compatible with clinical hypothyroidism (TSH 7.89 mUI/L; FT4 10.43 pmol/L) (Table 1). Following these findings, another neck US was performed, ultimately reporting the thyroid gland’s absence in its usual topography and the presence of a predominantly solid nodular formation in the submental region compatible with lingual ectopic thyroid tissue (Figure 4). A thyroid scintigraphy with Tc-99m pertechnetate confirmed an increased uptake in this submental region and an absence of uptake in the expected thyroid topography, thus supporting the suspicious diagnosis of ectopic thyroid (Figure 5).

After multidisciplinary review (by Endocrinology, Head and Neck Surgery, and Pathology), no further surgical procedure was considered. Further workup revealed negative thyroid autoimmunity, and levothyroxine supplementation was initiated one month after the second surgery, with thyroid function normalization (TSH 4.71 mUI/L; FT4 13.50 pmol/L) four months later (Table 1). The patient maintains regular follow-up in our outpatient Endocrinology clinic.

## Discussion

Ectopic thyroid is a rare occurrence but an important differential diagnosis to consider when approaching cervical masses, namely if a thyroglossal duct cyst is suspected. Of all ectopic thyroid glands, the majority are lingual (around 90%) and can manifest through upper aerodigestive tract symptoms (dysphagia, dysphonia, cough, and globus pharyngeus), though some patients remain asymptomatic [2]. In approximately 75% of lingual thyroid cases, this ectopic tissue is, in fact, the only functional thyroid gland, leading to an increased risk of hypothyroidism in these individuals [3]. No treatment is usually required in asymptomatic euthyroid patients, but regular follow-up is recommended to detect early cervical symptoms and prevent future complications [2,3,7,8]. On the other hand, thyroglossal duct cyst is a far more common condition (prevalence around seven in 100 individuals) [3]. As well as ectopic thyroid, it usually presents as an asymptomatic midline cervical mass and is frequently referred to surgical excision due to its aesthetic relevance or malignancy suspicion [3,4].

In our case, the first US report misled our management by describing a large cyst in the infrahyoid region as well as an eutopic, nevertheless small thyroid. This highlights the fact that, despite being the first-choice imaging technique for thyroid and neck evaluation, US presents relevant operator limitations. As an example, some studies have reported important intra and interobserver variations in the assessment of thyroid volume, with variabilities of around 14% and 17%, respectively [9]. Additionally, the operator’s experience is a well-known factor for correct US performance and evaluation, well described in some studies regarding thyroid nodules’ sonographic evaluation [10]. Human factor is especially present in US performance and interpretation; therefore, more than one imaging technique might be required in the diagnostic approach of some more difficult or inconsistent cases.

In addition, there was an inconsistency between the second neck US and CT findings. Neck US remains the gold standard imaging modality for thyroid evaluation; it is superior in assessing morphology, volume, and parenchymal texture, particularly in small or atrophic glands, owing to its high spatial resolution, real-time dynamic assessment, and ability to differentiate subtle changes in echogenicity and vascularization [11]. In contrast, CT presents limitations in detecting atrophic thyroid tissue when compared to US; it has reduced sensitivity for identifying thyroid abnormalities, as it relies on density and contrast differences that may be less evident in small-volume or atrophic glands [12]. In our case, the US report described the presence of an “atrophic eutopic thyroid”, whereas the CT report made no reference to this finding. Consequently, given the lower sensitivity of CT, greater weight was placed on the US findings, which influenced subsequent clinical management.

It is also important to note that all pathology reports are influenced by the clinical information provided; therefore, standardized details regarding the analyzed specimens should be provided, namely their location, clinical suspicion, and relevant laboratory assessments (such as thyroid function). In our case, the first pathology report described the presence of thyroid tissue, without the expected histologic criteria of a thyroglossal duct cyst (epithelial-lined cyst(s), sometimes adjacent to hyoid bone, and variable presence of thyroid tissue) [13]. The second pathology report, stating that the lesion removed on the second surgical intervention was compatible with follicular tumor of uncertain malignant potential of the thyroglossal duct, was based on the premise of a thyroglossal duct cyst excision, according to the clinical information provided, and reinterpreted as benign thyroid follicular nodular disease following recognition of thyroid ectopia. In both reports, thyroid tissue was identified, and the subsequent diagnostic reclassification reflected a correction of the initial clinical assumption.

Although thyroid autoimmunity status was unknown prior to the intervention, postoperative biochemical evaluation of the new-onset hypothyroidism revealed negative thyroid autoantibodies, thereby ruling out autoimmune thyroid disease as the most likely cause. Interestingly, the correct diagnosis of a sublingual ectopic thyroid was only established when, in a multidisciplinary setting, all imaging exams and pre and postoperative thyroid function were thoroughly reviewed, clearly highlighting the crucial role of multidisciplinary clinical discussion in every case.

The onset of hypothyroidism in previously euthyroid patients following a Sistrunk procedure for a suspected thyroglossal duct cyst has already been reported in the literature. Elechi et al. reported the case of a two-year-old boy who was submitted to this procedure and progressed to hypothyroidism postoperatively [14]. Similarly to our case, there was also a failure to identify the removed tissue as an ectopic thyroid, as preoperative US apparently documented the presence of eutopic thyroid lobes next to the suspected cyst. Nevertheless, most cases of sublingual or thyroglossal ectopic thyroid are correctly diagnosed before their removal, as the ones reported by Constantin et al. and Madana et al., in which untreated hypothyroidism was avoided by performing neck US, cervical magnetic resonance imaging (MRI)/CT, or thyroid scintigraphy in a timely manner before surgery [15,16].

Notably, thyroglossal duct cyst and thyroid ectopia might coexist. In a rare occurrence, Malek et al. described a 27-year-old male patient with a submental mass, whose neck CT found a submental cyst and a lingual thyroid with an absent eutopic thyroid [17]. Additionally, the confounding pathway between these diagnoses is also highlighted by Messias et al. in the case of a 41-year-old woman with a neck mass suggestive of thyroglossal duct cyst, but whose diagnostic workup (neck CT with contrast, thyroid scintigraphy, and fine-needle aspiration) ended up also revealing an ectopic thyroid [18].

**Figure 6:**
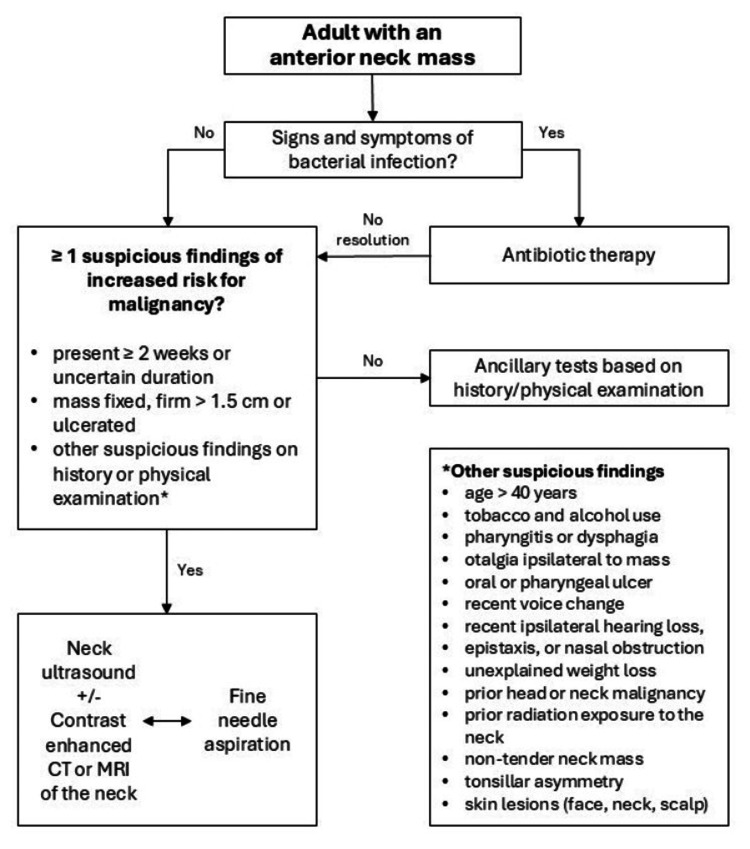
Algorithm of initial approach to an anterior neck mass. Adapted from: Pynnonen et al., 2017 [1]

There is still no consensus regarding the preoperative imaging approach to anterior neck masses (Figure 6). Some authors defend that neck US should be empirically performed in all patients preoperatively, particularly if a thyroglossal duct cyst is suspected, aiming to identify normal eutopic thyroid and rule out ectopic tissue, which in some cases might represent the only functioning thyroid [19]. Other cervical imaging techniques, such as CT or MRI, can also provide a better approach to neck masses, and if the hypothesis of ectopic thyroid tissue is raised by these exams, a thyroid nuclear scan should be performed to locate all functioning tissue [3]. On the other hand, other authors state that the low incidence of ectopic thyroid does not justify routine preoperative imaging, namely neck US, as it does not contribute to the optimization of the surgical plan in most cases [20].

## Conclusions

Our case adds to the rare reports of a sublingual ectopic thyroid mistaken for a suspected thyroglossal duct cyst. There is still no consensus regarding the preoperative imaging approach to anterior neck masses, but this report emphasizes the importance of an adequate case presentation and thorough laboratory and neck imaging evaluation in a multidisciplinary setting in every case prior to any thyroid-related surgical proposals. Consequently, this approach could reduce morbidity and improve clinical outcomes in these patients.
